# Fragmented Networks: Challenges in communication and cohesion of European Biodiversity Research Infrastructures

**DOI:** 10.3897/BDJ.13.e148079

**Published:** 2025-06-20

**Authors:** Allan T Souza, Tomáš Martinovič, Carrie Andrew, Yi-Ming Gan, Erik Kusch

**Affiliations:** 1 Institute for Atmospheric and Earth System Research INAR, Forest Sciences, Faculty of Agriculture and Forestry, University of Helsinki, Helsinki, Finland Institute for Atmospheric and Earth System Research INAR, Forest Sciences, Faculty of Agriculture and Forestry, University of Helsinki Helsinki Finland; 2 IT4Innovations, VSB – Technical University of Ostrava, Ostrava, Czech Republic IT4Innovations, VSB – Technical University of Ostrava Ostrava Czech Republic; 3 Natural History Museum, University of Oslo, Oslo, Norway Natural History Museum, University of Oslo Oslo Norway; 4 Royal Belgian Institute of Natural Sciences, Brussels, Belgium Royal Belgian Institute of Natural Sciences Brussels Belgium; 5 University of Oslo, Oslo, Norway University of Oslo Oslo Norway

**Keywords:** DiSSCo, eLTER, GBIF, LifeWatch ERIC, biodiversity, BioDT, Digital Twin

## Abstract

European Biodiversity Research Infrastructures (BioRIs) play a central role in addressing the complex challenges in biodiversity research, scientific collaboration across disciplines and national boundaries, as well as informing the public and policy-makers about the status and challenges of the European biodiversity. Our study focuses on the communication and coordination amongst BioRIs and revealed important fragmentation in communication strategies both within and across the key European BioRIs — including DiSSCo (Distributed System of Scientific Collections), eLTER (Integrated European Long-Term Ecosystem Research), GBIF (Global Biodiversity Information Facility) and LifeWatch ERIC. This fragmentation manifests in uneven geographical representation, inconsistent communication practices and limited data and service cohesion, ultimately impeding collaboration and the efficient use of resources. While some initiatives to tackle this issue demonstrate the potential for harmonisation, the the broader systemic challenges still persist. We argue that overcoming these barriers will require the development of standardised communication frameworks, more equitable distribution of infrastructures and deeper understanding of domain-specific differences that currently hinder the interoperability. Our study demonstrates the urgent need for coordinated efforts to integrate European BioRIs into a more coherent and accessible research ecosystem capable of addressing the biodiversity challenges of the 21^st^-century.

## Introduction

Research Infrastructures focusing on biodiversity (hereafter referred to as BioRIs) are facilities that provide data, analytical tools and collaborative platforms to support scientific communities in conducting research on biodiversity. A BioRI can be represented by a single site facility or a distributed network across multiple locations including major scientific equipment, collections, archives or scientific data, computing systems and communication networks (Hallonsten 2020, Fabre et al. 2021).

BioRIs provide important services to the scientific community, for instance, producing standardised methods, tools, data and platforms that facilitate the development of scientific research (Fabre et al. 2021). More importantly, they can facilitate access to standardised data across different regions, countries and continents, fostering the development of data intensive research (Farshidi et al. 2023). Such infrastructures are particularly relevant to the study of biodiversity patterns and processes, as there are still many gaps in knowledge, geographic coverage and implementation of standards in this field (Bach et al. 2012). In fact, our planet is currently experiencing an unprecedented crisis in biodiversity (Johnson et al. 2017). This crisis has several angles (e.g. climatic, pollution, deforestation, overexploitation) and affects different ecosystems and taxonomic groups in a multitude of ways (Dobson et al. 2021). BioRIs can support the scientific community to produce more complex and richer data outputs tackling these challenges (Farshidi et al. 2023).

Given the important role of BioRIs in advancing scientific knowledge, it is crucial that they remain well-connected within their own networks and their partners' networks. Well connected and informed BioRIs provide a more coherent platform for data access and sharing, reduce redundancy and enhance the ability to build on existing knowledge (Farshidi et al. 2023). Towards this goal, it is important to not only to focus on the technical interoperability, but also robust communication amongst the people who operate and use the BioRIs. Effective collaboration, with free and easy access to establish dialogue amongst BioRI staff, is essential for aligning goals, sharing best practices and ensuring that diverse RIs can work together efficiently to facilitate novel and relevant research impacts (Hodapp and Hanelt 2022).

**Figure 1.**
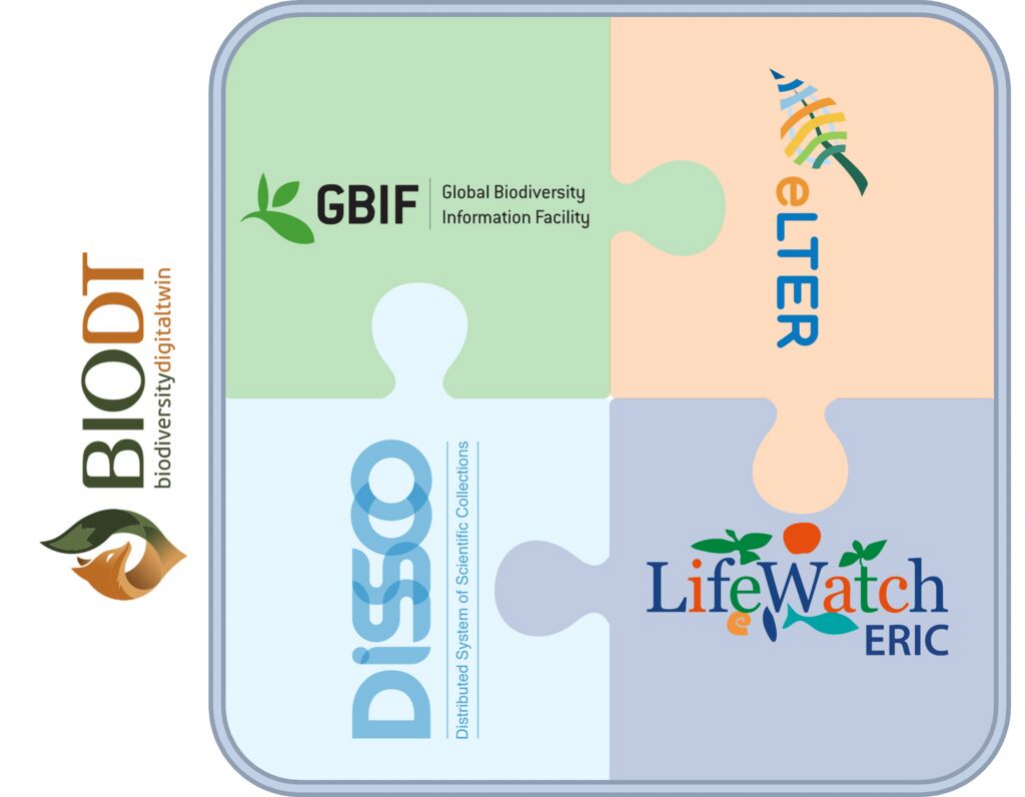
Conceptual representation of the Biodiversity Digital Twin (BioDT) with the integration of the four research infrastructures (DiSSCo, eLTER, GBIF and LifeWatch ERIC) involved in the development of the digital twin.

**Figure 2.**
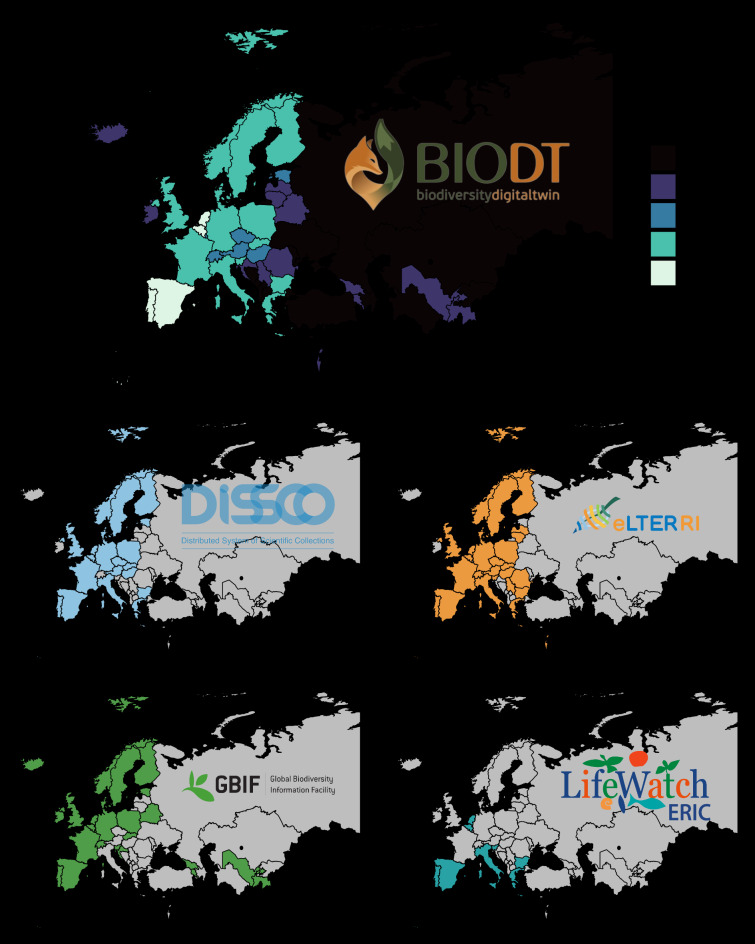
Distribution and representation of European Biodiversity Research Infrastructures is fragmented (A), with each individual research infrastructure differing in its geographical coverage (B).

An example of the benefits of such collaboration is the development of the Biodiversity Digital Twin (BioDT) project (Fig. 1). This project is facilitated through the services and resources offered through four distinct RIs that play pivotal roles in biodiversity research: Distributed System of Scientific Collections (DiSSCo), Integrated European Long-Term Ecosystem, critical zone and socio-ecological Research (eLTER), Global Biodiversity Information Facility (GBIF) and LifeWatch ERIC. Each of these distributed RIs (see Fig. 2 for an overview of their respective national representations) represents an international initiative varying in maturity, scope and areas of expertise, but together they form a comprehensive network that enhances our ability to understand and address biodiversity challenges (Table 1).

In this manuscript, we assess the capabilities of these four BioRIs (i.e. DiSSCo, eLTER, GBIF and LifeWatch) in facilitating scientific advancements. To this end, we investigate: I) the communication coordination efforts within and across the four BioRIs; II) the impact on the scientific frontiers of biodiversity-related research; and III) the cohesion and interoperability of their services and resources. Subsequently, we identify the existent strengths and challenges that the selected BioRIs operating in Europe have.

Finally, we provide a set of recommendations for how continued usefulness of these infrastructures can be maintained and how coordination of their efforts may be aligned better in the future, thus facilitating more cutting-edge research at European as well as global scales.

## Communication and Coordination Efforts of European BioRIs

Effective communication and coordination of RIs, particularly of distributed systems, has been identified as a major key performance indicator by funding bodies, research infrastructure managers, as well as end-users (Reid 2012, Kolar et al. 2019, Raes et al. 2020). However, communication and coordination are complex processes involving:

1. diverse sets of stakeholders,
2. means of sharing information, as well as
3. distinct pathways of information flow.

Analysing the state of the communication and coordination capabilities of European BioRIs thus necessitates investigation of all these three aspects simultaneously. Doing so enables subsequent recommendations of how to accomplish the key recommendation for clear channels of RI communications made by the European Commission (European Commission 2010).

### Stakeholders and Communication Participants

In assessing stakeholders and participants in a communication and coordination network comprised of several research infrastructures, there exist three distinct levels of communication and coordination efforts:

1. within a singular research infrastructure (i.e. information-flow between entities belonging to the same distributed research infrastructure);
2. between and across multiple research infrastructures (e.g. information-flow between an entity of GBIF and an entity of eLTER) and finally
3. between research infrastructures and the public (e.g. information-flow from within a research infrastructure to the public in response to an enquiry made by a member of the public).

The heterogeneous nature of maturity level, target audiences and services provided by the four target RIs (eLTER, DiSSCo, GBIF, LifeWatch) made it challenging to generate an objective, quantitative and standardised assessment of communication/coordination efficiency across these levels and audiences is challenging and, hence, it was not previously done.

To address this limitation in our current understanding of the communication and coordination capabilities of the four target BioRIs, we conducted a survey of research infrastructure managerial staff (i.e. with the national representative person for the RI). Within this survey (see Suppl. material 1), we requested that managerial staff of national representations (i.e. nodes/sites) of each target BioRI self-report their communication and coordination with other nodes belonging to the four target BioRIs. In designing this survey, we adhered to long-established best practices of survey design (Schaeffer and Dykema 2020) and solicited rankings of collaboration intensity across a Likert scale (ranging from 1 to 5) whose values correspond to:

1. Shared group calls or mailing lists;
2. Sporadic personal/one-on-one interaction;
3. Collaboration on shared projects;
4. Collaborate on shared tasks;
5. Shared offices and or staff.

By providing these set interpretations of the Likert scale intervals, we attempted to diminish the effect of subjectivity in responses given by BioRI managerial staff. Lastly, we also allowed a selection of "no interaction/collaboration" to denote instances of the BioRI network being entirely disconnected and without communication pathways or coordination efforts. Leveraging the BioRI-self-reporting of communication/coordination intensity, we indexed communication and coordination intensity: (1) within singular BioRIs and (2) across multiple BioRIs. Finally, the response rate to the survey itself is used as a proxy for: (3) communication intensity with members of the public.

**Figure 3.**
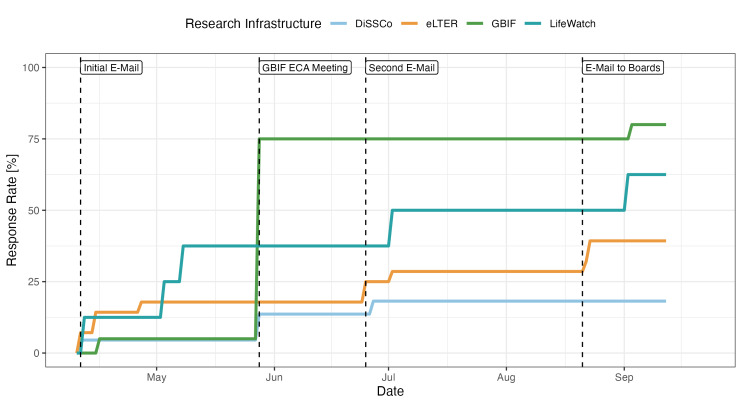
Survey responses of queried BioRIs. Plotting rate of response (% of queried national representations of each BioRI who responded to the survey inquiry) over time since first issuance of the enquiry reveals poor responsiveness across all target BioRIs with some BioRIs performing particularly poorly at communicating with the public. In addition, despite all BioRIs represented here being distributed systems, the value of in-person meetings (e.g. GBIF ECA) in soliciting information from within BioRIs remains invaluable.

In total, the survey was sent to 77 BioRI staff from the four target RIs (eLTER = 28, DiSSCo = 21, GBIF = 20 and LifeWatch = 8). The global response rate was 46.8% (n = 36), with GBIF staff having the highest response rate (80%), followed by LifeWatch (62.5%), eLTER (39.3%) and DiSSCo (19%), respectively. Assessing the communicativity of the European BioRIs with representatives of the public, we identified a regrettable and alarmingly low rate of responsiveness — both in rate-of-response (i.e. how many of the queried managerial staff responded to the survey issued) and time-to-response (i.e. time elapsed between issuance of enquiry and response rendered from the queried managerial staff). The response rates over time to our information-gathering survey can be found in Fig. 3. The GBIF's highest responsiveness to the survey is clearly linked to the advertisement for the survey amongst GBIF staff present at the in-person meeting of European and Central Asia nodes (GBIF ECA).

**Figure 4.**
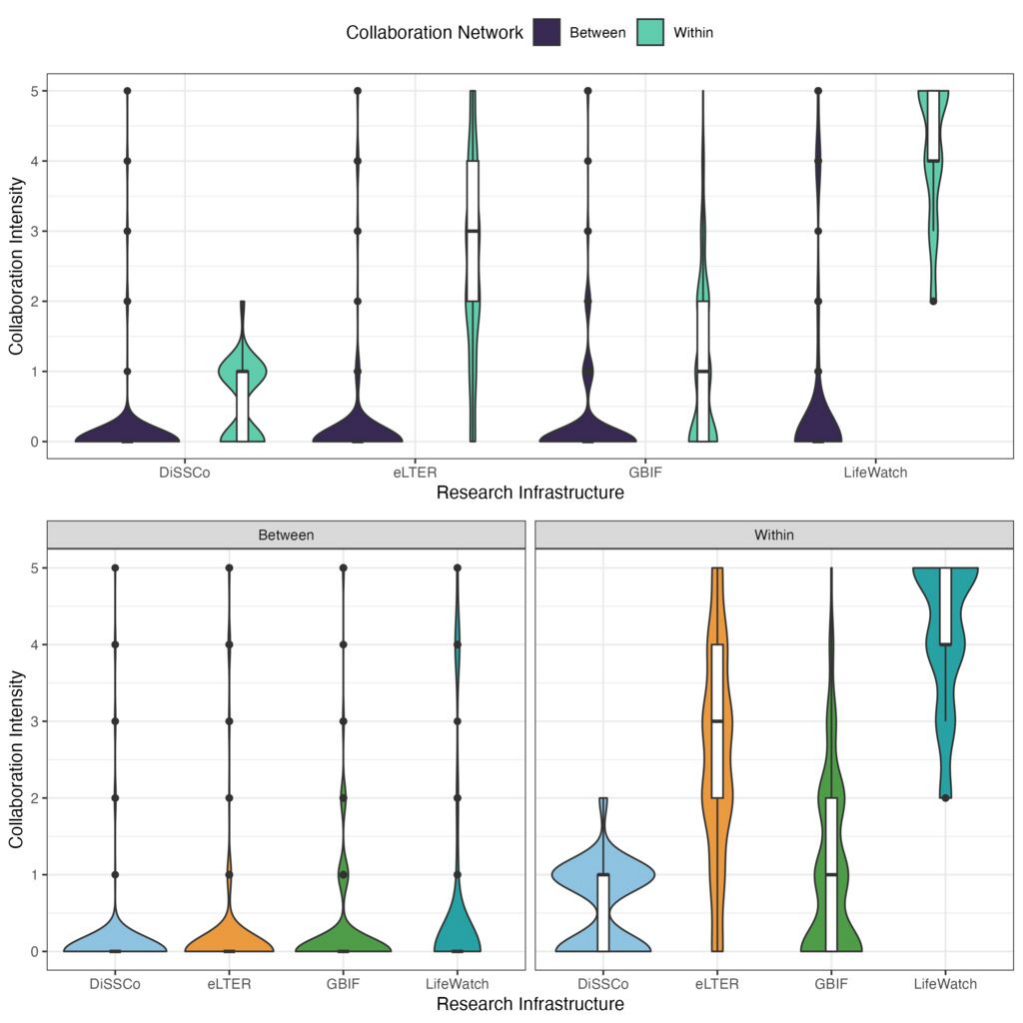
Self-reported collaboration intensity within and between four biodiversity research infrastructures (BioRIs). Upper panel: Collaboration of all BioRIs (DiSSCo, eLTER, GBIF and LifeWatch) are largely siloed, with minimal interaction across BioRIs being reported. Lower panel: Across BioRI, collaboration is uniformly low (left), while within BioRI, collaboration varies, with LifeWatch and eLTER showing the highest intensity (right). These patterns highlight limited cross BioRI integration and variable internal cohesion, highlighting the fragmented scenario in the BioRI landscape.

The responses to our survey also identified a clear trend of communication and coordination being stronger within each BioRIs and much weaker across BioRIs (Fig. 4). While this should be expected — for several reasons including alignment of topics, shared vocabularies, as well as shared means of communications within each BioRI — it is nevertheless concerning that most national BioRI representations hardly interact at all with individual national representations of a different BioRI. While the four target BioRIs perform similarly poorly at communicating with entities of other BioRIs, there are pronounced differences of collaboration intensity within singular BioRIs, particularly, eLTER and LifeWatch RIs collaborate much more intensely within their RIs than DiSSCo and GBIF do within theirs. It is difficult to pinpoint the reasons for this difference, as no specific question targeting this issue was included in the survey; however, potential explanations for the higher degree of internal interaction showed by eLTER and LifeWatch could be linked to higher team cohesion; specific project/institutional management practices; and the existence of dedicated and well functioning channels for communication amongst the community (e.g. eLTER Sites and Platform Forum — https://elter-ri.eu/elter-plus/sites-and-platforms-forum; LifeWatch community — https://community.lifewatch.eu/).

While a multitude of competing and complementary key performance indicators have been proposed to evaluate communication and collaboration performance of BioRIs (Kolar et al. 2019), our results already indicate the need for improvement in communication and collaboration within and across BioRIs. Our results do not cover the full spectrum of the BioRIs' communication and coordination efforts, but identifies particularly relevant communication levels across which BioRIs perform differently. This result suggests that further and more detailed investigation for reasons behind differing levels of communication within BioRIs is needed and that the good examples from other BioRIs could be used to develop recommendations and guidelines for remedying shortcomings in one BioRI by learning from the processes enacted in another.

### Means of Communication

Sharing of information can be carried out via a multitude of means (e.g. personal communication, mailing lists, webpage, newsletters, social media) — each presenting distinct benefits and downsides for the purpose of communication with a specific stakeholder group. Throughout our interactive process of approaching and discussing with BioRI managerial staff for self-reports of realised communication and coordination potential, we also engaged in exhaustive conversations and exploration of which means of communication the different BioRIs frequently use. We found no clear alignment across or within BioRIs, as to which means of communication is preferably used for sharing of information within the scientific community.

When looking at the official documents from the BioRIs focusing on dissemination and communication, the contents reveal commonalities and particularities employed by the target BioRIs. For instance, the four BioRIs have formalised mechanisms for internal coordination, such as newsletters, virtual meetings or centralised project platforms, although the degree of detail on the tools and strategies used varies amongst them, with DiSSCo and eLTER explicitly listing tools such as dedicated intranets or training portals, while LifeWatch’s documentation refers only broadly to internal communication efforts without naming specific tools. Externally, all BioRIs mention a complex multi-channel dissemination approach, combining webpages, social media and public engagement events. DiSSCo and eLTER offer a broad toolkit of media assets and stakeholder-targeted outreach activities, whereas GBIF emphasises network-wide communication facilitated by its global node structure. LifeWatch, by contrast, highlights formalised public engagement through conferences and recognition programmes. These patterns suggest a common emphasis on multi-channel outreach, but also highlight that the BioRIs do not follow a harmonised plan for communication both within and across RIs (Table 2).

This absence of clear knowledge of which means of communication to engage with to obtain relevant information from BioRIs is exacerbated further by drawbacks of the most frequently used means of communication across our four target BioRIs. Firstly, email and mailing lists, while used almost ubiquitously, are private by design (email) and very difficult to spontaneously access (mailing lists) or stay up-to-date with their developments depending on the number of participants in the list. Secondly, discussion boards make for much easier gaining of an overview of information pertaining to topics of interest, but may not be desired to be made public by BioRI coordinators for data protection reasons and become confusing for the purpose of across-BioRI information sharing when different layouts and terminology are employed. Thirdly, social media platforms arguably combine the worst aspects of both mailing lists and discussion boards in that social media platforms usually make follow-up on past information impossible unless it was shared publicly in the first place and may preclude full access to relevant information through their compartmentalisation of communication into granular chat and group environments. Lastly, some national representations of research infrastructures rely heavily on information sharing via in-person communication such as workshops and conferences, as well as what can best be described as “oral tradition” — a means of communication not befitting the sharing of information outside a small group of people.

Ultimately, we find that, to realise more of the untapped communication and coordination potential within and across European BioRIs, a unifying means of communication is required which streamlines and simplifies information sharing through:

1. persistence and traceability of information;
2. standardised layouts and terminology and
3. classification of information as either public or private with designated stakeholder audiences.

### Directionality of Communications

There are two main types of communication engagement between research projects and stakeholders. One-way communication refers to the asynchronous exchange of information in time and space, such as publications, databases, newsletters, websites etc. Two-way communication involves real-time interaction between different parties, whether in-person or virtual, such as during conferences, workshops or meetings (Jolibert and Wesselink 2012). While the former directionality is comparatively easier to achieve and likely sufficient for most interactions and use-cases of the public with regard to BioRIs, two-way communication is crucial to improving coordination of European biodiversity BioRIs.

### Cohesion and Interoperability of Services and Resources

Biodiversity data standards originated as a computational offshoot of the data digitisation process, in that the process allowed for the accumulation of datasets into single repositories, which required the data to be standardised. Without data standards, biodiversity data lack the interoperability needed for consistent interpretation, compilation and integration. In addition, machine readability of data, crucial for research infrastructures as well as for AI, depends upon data standards for interoperability.

There are many forms of data standards that are critical for biodiversity data and BioRIs. Andrew et al. (2024) provide a more comprehensive guide to understanding biodiversity data standards. For our purposes, the important point is that data standards bring information into a standardised format for storage and that they support computational communication.

Biodiversity data standards were earliest applied to museum data (Graham et al. 2004) and soon after integrated into research data infrastructures like GBIF (Wieczorek et al. 2012, Robertson et al. 2014, D et al. 2017). With more forms of data represented in a greater number of infrastructures, the practicalities of data standardisation are changing (Schneider et al. 2019). One impediment is that standards have typically been created on an ad-hoc basis. Hence, the protocols and methods for their development vary and the terms they include are never comprehensive for all forms of biodiversity data (Andrew et al. 2024).

The four BioRIs that we focus on all contain different forms of biodiversity data and this reduces data interoperability due to low the amounts of shared data standards that they share (Andrew et al. 2024). Thus, in addition to our survey results demonstrating low communication between personnel of the different RIs (Fig. 4), the likelihood for data interoperability, through shared data standards, is also likely limited. GBIF and DiSSCo share the most data standards, because both contain museum data. Each apply the Access to Biological Collection Data (ABCD) and Darwin Core (DwC) data standards (Holetschek et al. 2012, Wieczorek et al. 2012, Walls et al. 2014). They also implement other data standards that are unique to each: Collection Descriptions (CD) and the Minimum Information about a Digital Specimen (MIDS) for DiSSCo and the Humboldt Core Extension (https://eco.tdwg.org; Guralnick et al. (2017)) for GBIF. In contrast, eLTER data come from site-level field data (Wohner et al. 2022) and are more heterogeneous. They cover datasets in the atmospheric, ecological, geological, hydrological and social sciences; it would be exceptionally challenging to standardise data across all of those topics. Instead, eLTER has created standards observations that emphasise the standardisation of measurements and protocols, rather than of variables between datasets. eLTER does not, then, overlap with any other RI in their data standards. They do support, but not require, the use of existing established vocabularies, such as the Environmental Thesaurus (EnvThes; Schentz et al. (2013)). The final BioRI under investigation here, LifeWatch, lacks any specific data standards because they do not directly aggregate data. Instead, LifeWatch mostly provides support for data logistics. LifeWatch does provide resources for applying data standards, such as EcoPortal (https://ecoportal.lifewatch.eu), which contains around 30 data standards and related domains, including the eLTER-supported EnvThes. Despite the differences in data, they all ultimately support biodiversity data. Hence, greater interoperability between datasets should conceivably increase communication between the RIs, in addition to providing greater likelihood of supporting the use of their combined data in research projects such as the Biodiversity Digital Twin (Trantas et al. 2023, Westerlaken 2024).

## Future Directions for Communication and Coordination of European BioRIs

### Common Communication Interface

Given the importance of communication and coordination amongst BioRIs, it is important to look to the future and envision solutions that can be developed to facilitate the exchange and availability of information to the BioRI community. This solution/tool should be able to address the community needs and facilitate the communication via chat, email, discussion boards, synchronised events calendar and social media.

Beyond these immediate features, the community would also benefit from considering more advanced requirements. These might include integration with code bases and issue tracking systems, as used in Git repositories, as well as connections with European Data Spaces and similar initiatives. While these possibilities are highly valuable, they introduce a level of complexity that goes beyond the scope of the current study. For now, we will avoid going too far down that path, but it is important to keep these needs in mind when evaluating long-term solutions.

One of the simplest approaches would be to use established tools to create common mailing lists, chat groups with services such as Slack or its European counterpart such as Stackfield, Fleep, create discussion board with tools like Lemmy, social media network on Mastodon and calendar in one of these tools. A disadvantage of such a solution is still having many tools, which are maintained by others and also the need to pay for usage of such services. Advantage is that these are usually working solutions with their own development and operation teams.

A slightly more involved approach would be to self-host many of these tools. Self-hosting a social media network should not be included in this approach, as that might be the smallest problem in terms of communication fragmentation we are focusing on. However, self-hosting other components like chat, email and calendars could offer more control and flexibility. This option comes with additional costs for developing and maintaining the services in long-term, which should be taken into consideration in strategic planning.

**Figure 5.**
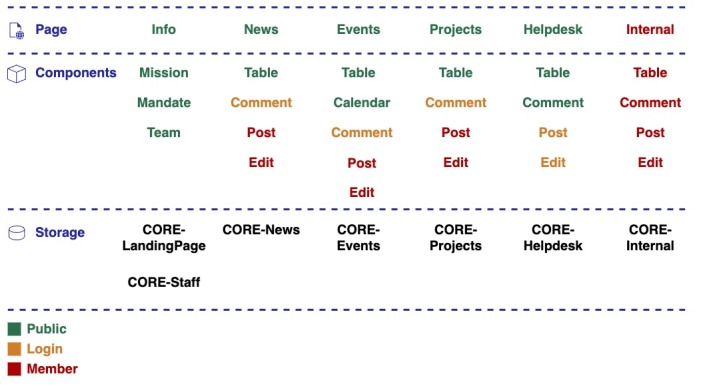
Envisioned solution/tool architecture for the Biodiversity Research Infrastructures (BioRIs) communication and coordination, showing pages in columns and individual components of the solution/tool. Green components are publicly available, orange one are available to everyone after they login to the system and red ones are available only to the members of the given group. At the bottom are Github repositories where the relevant information for given web page are stored.

The third option is to find a single software solution which will handle chat, email, discussion board and calendar (Fig. 5). There is a lot of different frameworks which covers these requirements; however, they usually also contain enormous amount of additional features which are not needed, increasing the complexity of the systems. Under this category, we could find business orientated systems like Odoo or collaboration system like Nextcloud Hub. An advantage of these is that, instead of multiple points of entry and multiple systems to operate, there would be a single system, potentially reducing costs on operation.

### Adoption and Operation Challenges

In the previous subsection, we introduced different approaches for the creation of common communication interfaces for the BioRIs. Here, we will discuss what are some of the challenges for the adoption of these solutions. We will start with challenges which are common for any of these solutions and continue with a short description of specific challenges of individual solutions. As this is a very complex topic, we will focus on major issues without going too deeply into technicalities.

The obvious challenge in creating a common system amongst BioRIs or, in essence, any large organisations are questions of funding, responsibility, governance and operation. There are many potential models which could be used to solve these and usually it would mean there would require creation of new positions to coordinate such efforts, thus increasing costs for each individual RI involved. One potential solution to minimise issues related to the cross-infrastructure problem would be to have one of the RIs take a lead and be responsible for managing the whole system, where other RIs would be the users of the system. This RI would then have a contract most likely with the EU governing bodies to create a central communication system for other BioRIs, assuming there would be willingness to fund such a project. This would simplify questions of responsibility, governance and operation; however, it would most likely not answer the question of funding, especially if the other RIs would have specific requirements such as addition of new features to the system. Still, such tasks could be part of the contract for the responsible RI or it could be covered by joint grant projects which would cover costs beyond the original contract. Of course, this solution would have to be accepted by other BioRIs and might lead to creation of a system that nobody will use in the end, since there might be a smaller incentive for adoption by the other RIs. On the other hand, if multiple RIs, or teams at RIs, would start using such a system and show its benefit, most likely, others would be drawn to it naturally. To increase incentives, a part of the contract would naturally need to cover step by step onboarding of teams from each RIs which could then bring the rest of the RI to the system. In essence, this is a model used by most of the new technological solutions entering the market and we can see that a similar concept is already being used with projects like Destination Earth, although, due to the scale of the project, there are multiple responsible organisations behind it.

Considering the specifics of the solutions mentioned beforehand, there are some small things to consider. Usage of existing tools hosted in Europe might be a good thing since it would support European businesses and most of them are compliant with the necessary European regulation which could reduce worries about the data privacy and so on. The need to pay for the services may not be really a negative thing, since with other solutions, costs of having people managing the operation of self deployed services is usually much larger, but this may depend on the scale of the solution. The possible risk in these cases might be that those services will change terms of use or cease to exist and the migration may be quite costly.

Using either multiple self-hosted open-source solutions or single self-hosted solution, the advantage would be that there would be a higher level of clarity about the data storage and potentially higher confidence in the service regarding privacy. In case of this solution, risks of the service operation changes would be minimized, since then they could be bound to the contract regarding the operation of the service by selected RI and even in case of existential problems of given RI, there should be a possibility of migrating this responsibility to another. Of course, there is still a risk that the open-source project which is being used for this solution will stop being maintained what would lead to questions about how to either migrate to a new solution or whether it is possible to take over maintenance of a given project, potentially increasing the costs and naturally transferring to solution number four.

Finally, we should consider creating a custom solution to cover the BioRIs communication needs. Such a solution would be the most beneficial from the long-term perspecive as it would give us total control over it. Since creating something new is a considerable challenge an easier way, often seen in software development nowadays, would be to use already existing open-source solution and adjust it to the specific needs of BioRIs. Using an open-source project with a large community would allow to divide the development responsibilities to the community minimising the challenges and costs, while maximising impact. Creation of the completely new solution might be appealing and maybe it would be necessary in the long run, but starting with existing solutions would make it possible to tackle this problem much faster.

Finally, we should consider creating a custom solution to address the BioRIs' communication needs. As is evident from the previous paragraph, a custom solution could be desirable in the long term. We suggest beginning with the second or third solution and gradually integrating into the development of the system. For instance, we could start by creating custom extensions to facilitate specific BioRIs' needs. This approach appears to be a sustainable way of addressing the issue while minimising friction in the adoption process. Eventually, we could become maintainers or co-maintainers of this solution. A key advantage of large open-source projects is that a community often exists to help ensure the product's vitality, as there are multiple user groups. While creating a completely new solution might be appealing and perhaps necessary in the long run, starting with existing solutions would enable us to tackle this problem much faster.

## Discussion

Our investigation of European Biodiversity Research Infrastructures (BioRIs) highlights considerable fragmentation in communication strategies both of within and across four distinct European BioRIs (i.e., DiSSCo, eLTER, DiSSCo, GBIF and LifeWatch). Such fragmentation presents significant challenges that hinder efficient collaboration, resource sharing and, ultimately, slows down scientific progress through misallocation of efforts and funds. These challenges are particularly evident in several key areas: geographical representation, communication and coordination, scientific areas impacted and data and service cohesion.

Despite one or more BioRIs being present in almost every European country, currently, BioRIs are unevenly distributed individually across Europe. This geographical fragmentation of individual BioRIs leads to unequal opportunities for researchers in different regions. To ensure that research infrastructures are accessible to all and avoid biases in research outcomes, there is a need for a more homogeneous representation of BioRIs across Europe. This would ensure that regions currently under-represented are provided with the same opportunities for scientific collaboration and resource access as more established regions. A more concrete effort to expand and balance BioRI representation could contribute to a more equitable research environment. This, however, is a costly undertaking which is unlikely to be fulfilled/realised without a coordinated funding effort and major influx of monetary resources. Ultimately and instead of a sudden change to ongoing international efforts to support and grow BioRIs, overcoming geographical fragmentation will likely and more feasibly be remedied better through increased communication and capacity sharing efforts across existing BioRI networks.

Such a need for increased communication and capacity sharing efforts is already supported by our findings. We find that BioRIs across Europe are fragmented in regard to ongoing communication and coordination across each other and perform differently at communicating and sharing efforts within themselves. Differing means of communication, most of which are geared towards one-way flow of information, are likely at the core of this siloed nature of BioRI communication networks. To overcome this limitation, which we find to be central to many issues faced by BioRIs as well as their user-bases, we recommend the creation and adoption of a standardised communication framework. By implementing such a system, BioRIs can strengthen internal collaboration, promote new partnerships and make information more accessible to the broader scientific community and the public. This would also help mitigate information silos and enhance stakeholder engagement.

Some efforts are already underway to address these communication challenges. For example, initiatives like the EU-funded GLOBIS-B project have demonstrated the potential for successful collaboration across geographically and thematically diverse RIs. GLOBIS-B has focused on mobilising data and generating knowledge across multiple domains, organising workshops that unite scientific communities and service providers under common objectives (Kissling et al. 2015). Another example is the ENVRIplus project, which has worked to harmonize observation methodologies and access policies across multiple European Environmental and Earth System RIs. This effort has fostered collaboration through staff exchange programmes and the generation of common solutions for shared infrastructures (ENVRIplus 2024). The ENVRI Hub (https://envrihub.vm.fedcloud.eu/) is a unifying platform that connects Europe’s environmental and biodiversity research infrastructures into a single, accessible hub. It enhances collaboration by breaking down silos between different RIs and centralising data and services, which makes scientific knowledge more readily available.

These examples highlight the importance of robust communication strategies. Previous research has shown that strong, user-friendly communication platforms can significantly enhance collaboration amongst RIs (D’Ippolito and Rüling 2019, Balli et al. 2022). Developing such tools, along with creating spaces for regular inter-RI meetings and collaborative online environments, would foster a sense of community and shared purpose. This can also lead to more efficient problem-solving and innovation, as researchers and infrastructure providers can exchange ideas and experiences more freely. Open dialogue can help RIs align their development efforts and streamline their approaches towards achieving common goals. Achieving the ambitious demands of cutting-edge research to address biodiversity issues of the 21^st^ century will depend on the development of seamless communication policies and tools that enable different infrastructures to integrate their data and services efficiently (Trantas et al. 2023, Westerlaken 2024).

While the diversity of research areas impacted by different BioRIs is a valuable asset poised to respond the research demands of the 21^st^ century, it underscores the importance of data cohesion and compatibility. One ongoing concern is to what extent the lack of communication between BioRIs is due to systemic differences in their objectives and the types of data they handle. A better understanding of these underlying differences is needed to assess whether they contribute to fragmentation or if they could be harmonised to enhance interoperability. We suggest that this deeper understanding can only be created iteratively through efforts like literature tracking services already implemented in some BioRIs we have investigated. Subsequently, addressing these issues could pave the way for more comprehensive and coherent data integration, benefitting the entire biodiversity research landscape.

In conclusion, tackling fragmentation within European BioRIs requires a multi-faceted approach. Ensuring broader geographical representation, fostering collaboration through standardised communication platforms and promoting data cohesion will be key steps towards a more integrated and efficient research infrastructure network. These efforts will ultimately support more innovative and impactful biodiversity research, ensuring that Europe remains at the forefront of global efforts to understand and protect biodiversity.

### Limitations of the study

While our investigation offers important insights into the communication challenges and structural fragmentation of European BioRIs, it is important to note that the study has some limitations that must be acknowledged. First, our analysis focused primarily on a select subset of BioRIs (targeted to BioDT consortium — DiSSCo, eLTER, GBIF and LifeWatch) and may not capture the full diversity of environmental and biodiversity research infrastructures operating across Europe. Second, much of the data analysed was derived from a target survey, publicly available communication materials, strategic documents and websites, which may not fully reflect the internal coordination mechanisms or informal practices that shape collaboration within and across BioRIs. It is also important to highlight that this study basically relied on self-reported qualitative assessments, with a relatively small sample size (only representatives of the BioRIs) and did not account for the entirety of the BioRIs community. Moreover, given the dynamic nature of funding mechanisms, partnerships and research infrastructure development, our findings represent a temporal snapshot that may not account for ongoing or recent shifts in strategy. Future research would benefit from more systematic, longitudinal data collection, including interviews and surveys with BioRI stakeholders, to build a deeper and more nuanced understanding of how communication and integration efforts evolve over time.

## Data Availability Statement

All data and code supporting this manuscript are available at https://github.com/ErikKusch/BioDT-Research-Infrastructure.

## Supplementary Material

9A27E9D8-FA76-5066-9A87-280C8F876A78 10.3897/BDJ.13.e148079.suppl1 Supplementary material 1 Research Infrastructure Communication Survey Data type Survey Form Brief description A PDF of the Google form used to solicit self-reported metrics of communication and collaboration within and between RIs. File: oo_1131457.pdf https://binary.pensoft.net/file/1131457 Erik Kusch, Allan T. Souza

## Figures and Tables

**Table 1.** Key characteristics of the biodiversity research infrastructures under investigation.

**RI**	**Started year**	**Current number of member countries**	**Coverage**	**Main aim**	**Spheres of knowledgement covered**	**Status**	**Key products and/or tools**	**Data services maturity**
DiSSCo	2018	23	European	To digitise and mobilise data from natural history museums	Biosphere	Project phase	Digitisation tools, data portals	Under development
eLTER	2020	26	European	To facilitate long-term ecological observations and research across various European ecosystems	Atmosphere, Geosphere, Hydrosphere, Biosphere and Sociosphere	Project phase	Long-term ecological observations data	Under development
GBIF	1999	45	Global	To provide free and open access to biodiversity data on a global scale	Biosphere	International organisation	Occurrence data, Species checklists, Event data, data publishing tools	Established
LifeWatch	2006	8	European	To provide advanced e-Science research facilities for biodiversity and ecosystem studies	Biosphere	ERIC (European Research Infrastructure Consortium)	Virtual research environments (VREs), modelling tools	Established

**Table 2.** Communication strategy of the four Biodiversity Research Infrastructures (BioRIs), detailing the strategies employed for internal and external communication.

**Research Infrastructure**	**Internal communication**	**External communication**	**Source**
DiSSCo	- Slack (team messaging) - Teamwork (project intranet platform) - Email (internal mailing communications) - Regular virtual meetings (online project meetings) - Internal newsletters (project updates for partners)	- Webpage - Social media (official DiSSCo accounts on Twitter, Facebook, LinkedIn, Instagram) - Press releases and media (public news releases) - Printed/media materials (brochures, posters, images, videos) - Stakeholder events (roundtables, workshops, webinars, virtual events for outreach)	Casino et al. (2020)
eLTER	- Webpage - “Training Corner” on website (for internal training resources) - Internal newsletters (bi-annual internal updates for network members) - Quarterly project newsletter (regular newsletter produced by eLTER) - Promotional materials (brochures, posters, stickers) - Factsheets (internal progress highlights) - Press releases (project news prepared for media) - Project events (internal trainings, workshops, summer schools etc.)	- Social media (eLTER’s external Facebook & Twitter channels) - Public e-newsletter (quarterly newsletter for external audiences; also shared via partner organisations’ websites/newsletters) - Specialised outlets (industry, environmental or policy magazines and channels) - Scientific publications (papers in journals, including data papers) - Mass media outreach (press coverage in general media)	Demirova et al. (2025)
GBIF	- Participant node communications (regular internal updates to GBIF Nodes via mailing lists or similar channels) - Webinars for network (GBIF Secretariat webinars to Node managers) - Internal newsletters (communications from Secretariat to network members)	- Webpage - Social media (GBIF official social media platforms) - Email newsletters (opt-in general newsletter; dedicated publisher newsletter) - Community forum (online discussion platform for the GBIF community)	GBIF Secretariat (2015)
LifeWatch ERIC	No specific tools are detailed in the strategy document.	- Webpage - Printed publications (corporate brochures and other printed outputs) - User conferences (periodic LifeWatch ERIC user conferences for community engagement)	Arvanitidis et al. (2024)
