# Supplementary material for: Fragmented Networks: Challenges in communication and cohesion of European Biodiversity Research Infrastructures
Source: Biodivers Data J. 2025 Jun 20;13:e148079. doi: 10.3897/BDJ.13.e148079 (PMC12238962; doi:10.3897/BDJ.13.e148079)
Supplement: Supplementary material 1 — Research Infrastructure Communication Survey [file bdj-13-e148079-s001.pdf]

BioDT Research Infrastructure Cooperation Survey

The biodiversity digital twin project (BioDT) is set to develop a number of prototype digital twins to help protect and restore biodiversity across Europe and beyond. This work is predicated on data and services mediated through European Biodiversity Research Infrastructures (RIs) like (1) the Distributed System of Scientific Collections (DiSSCo), (2) the Integrated European Long-Term Ecosystem, critical zone and socio-ecological Research (eLTER), (3) the Global Biodiversity Information Facility (GBIF), and (4) LifeWatch ERIC, all of which are partners in BioDT.

Much of cutting edge ecological research to address the challenges of the Anthropocene could not be carried out without these and other RIs. However, our understanding of the European biodiversity RI remains lacking on several levels (e.g.; managerial, data exchange, user-interaction, etc.). As part of work package 4 within BioDT, we aim to address this knowledge gap and subsequently provide guidance and principles of RI management and collaboration.

In this survey, we want to poll information of how strongly nodes/representatives of different RI networks and nationalities interact with each other. We will use this information, anonymised, to facilitate a graph analysis and subsequent report on the current state of the European biodiversity RI landscape. The survey below has three sections:

1. Consent declaration and representation attributes
2. Collaboration with BioDT consortium RIs
3. Collaboration with non-BioDT consortium RIs

Responding to the mandatory sections 1 and 2 should take approximately 5 minutes of your time while we invite you to spend some more time, as you have available, on the optional section 3 to deepen our understanding of the RI network beyond the 4 BioDT consortium RIs. We appreciate the time that you can take to fill out this survey.

Please notice that **information supplied by you will be treated confidentially and anonymized before analysis**. How do we do this? Each response will be in relation to another research infrastructure node/site and thus mirrored by another respondents entry. For analysis, these entries will be compiled to mean values and so not be able to be traced back to individual persons.

Should any questions arise throughout this survey, please do not hesitate to direct them to erik.kusch@nhm.uio.no.

* Indicates required question

1. I understand that the data I provide will be used for research purposes and may support peer-reviewed publications for which the data I supply will be made publicly available following anonymisation procedures. *

Tick all that apply.

- [ ] Yes, I acknowledge and give consent.

2. Which Research Infrastructure do you represent? *

Mark only one oval.

- ( ) DiSSCo
- ( ) eLTER
- ( ) GBIF
- ( ) LifeWatch

3. Which nationality/country is your specific research infrastructure node/site based in? *

____

Interaction with **BioDT Consortium** Research Infrastructures

In this section, we are interested in how closely your national research infrastructure node/site (i.e., you and/or your team) collaborate with nodes/ representatives of DiSSCo, eLTER, GBIF, and LifeWatch.

We ask you to rate your collaboration intensity as either "No Interaction" (you have no contact with the node/site of the RI and nationality in question) or on a likert scale ranging from 1 to 5 to indicate how closely you collaborate with the RI node/site/ representative in question.

To avoid confusion of how to work with the 1-5 scale, here are some suggestions for how to link your collaboration to the levels in the scale:

1. You are present in group calls or mailing lists with the node/site in question
2. You interact with staff of the node/site in question sporadically
3. You collaborate on projects with the node/site in question
4. You collaborate on shared tasks with the node/site in question
5. You share offices and or staff with the node/site in question

NOTE: If you are a BioDT project partner, please answer as though you were not to avoid a strong biasing effect of this project on our understanding of the day-to-day makeup of the European biodiversity RI landscape.

4. Please rate how closely you work with the following **DiSSCo** nodes. *Please make only one selection per row.* *

Tick all that apply.

	No Interaction	1	2	3	4	5
**Austria**	☐	☐	☐	☐	☐	☐
**Belgium**	☐	☐	☐	☐	☐	☐
**Bulgaria**	☐	☐	☐	☐	☐	☐
**Czech Republic**	☐	☐	☐	☐	☐	☐
**Denmark**	☐	☐	☐	☐	☐	☐
**Estonia**	☐	☐	☐	☐	☐	☐
**Finland**	☐	☐	☐	☐	☐	☐
**France**	☐	☐	☐	☐	☐	☐
**Germany**	☐	☐	☐	☐	☐	☐
**Greece**	☐	☐	☐	☐	☐	☐
**Hungary**	☐	☐	☐	☐	☐	☐
**Italy**	☐	☐	☐	☐	☐	☐
**Luxembourg**	☐	☐	☐	☐	☐	☐
**Netherlands (Head Office)**	☐	☐	☐	☐	☐	☐
**Norway**	☐	☐	☐	☐	☐	☐
**Poland**	☐	☐	☐	☐	☐	☐
**Portugal**	☐	☐	☐	☐	☐	☐
**Slovakia**	☐	☐	☐	☐	☐	☐
**Spain**	☐	☐	☐	☐	☐	☐
**Sweden**	☐	☐	☐	☐	☐	☐

**United Kingdom**	☐	☐	☐	☐	☐	☐
**Switzerland**	☐	☐	☐	☐	☐	☐

5. Please rate how closely you work with the following **eLTER** nodes. *Please make only one selection per row.* *

Tick all that apply.

	No Interaction	1	2	3	4	5
**Austria**	☐	☐	☐	☐	☐	☐
**Belgium**	☐	☐	☐	☐	☐	☐
**Bulgaria**	☐	☐	☐	☐	☐	☐
**Czech Republic**	☐	☐	☐	☐	☐	☐
**Denmark**	☐	☐	☐	☐	☐	☐
**Finland**	☐	☐	☐	☐	☐	☐
**France**	☐	☐	☐	☐	☐	☐
**Germany (Head Office)**	☐	☐	☐	☐	☐	☐
**Greece**	☐	☐	☐	☐	☐	☐
**Hungary**	☐	☐	☐	☐	☐	☐
**Ireland**	☐	☐	☐	☐	☐	☐
**Italy**	☐	☐	☐	☐	☐	☐
**Latvia**	☐	☐	☐	☐	☐	☐
**Lithuania**	☐	☐	☐	☐	☐	☐
**Luxembourg**	☐	☐	☐	☐	☐	☐
**Netherlands**	☐	☐	☐	☐	☐	☐
**Norway**	☐	☐	☐	☐	☐	☐
**Poland**	☐	☐	☐	☐	☐	☐
**Portugal**	☐	☐	☐	☐	☐	☐
**Romania**	☐	☐	☐	☐	☐	☐

**Serbia**	☐	☐	☐	☐	☐	☐
**Slovakia**	☐	☐	☐	☐	☐	☐
**Slovenia**	☐	☐	☐	☐	☐	☐
**Spain**	☐	☐	☐	☐	☐	☐
**Sweden**	☐	☐	☐	☐	☐	☐
**Switzerland**	☐	☐	☐	☐	☐	☐
**Turkey**	☐	☐	☐	☐	☐	☐
**United Kingdom**	☐	☐	☐	☐	☐	☐

6. Please rate how closely you work with the following **GBIF** nodes. *Please make only one selection per row.* *

Tick all that apply.

	No Interaction	1	2	3	4	5
**Andorra**	☐	☐	☐	☐	☐	☐
**Belgium**	☐	☐	☐	☐	☐	☐
**Switzerland**	☐	☐	☐	☐	☐	☐
**Germany**	☐	☐	☐	☐	☐	☐
**Denmark (Head Office)**	☐	☐	☐	☐	☐	☐
**Estonia**	☐	☐	☐	☐	☐	☐
**Spain**	☐	☐	☐	☐	☐	☐
**Finland**	☐	☐	☐	☐	☐	☐
**France**	☐	☐	☐	☐	☐	☐
**Croatia**	☐	☐	☐	☐	☐	☐
**Ireland**	☐	☐	☐	☐	☐	☐
**Iceland**	☐	☐	☐	☐	☐	☐
**Luxembourg**	☐	☐	☐	☐	☐	☐
**Netherlands**	☐	☐	☐	☐	☐	☐
**Norway**	☐	☐	☐	☐	☐	☐
**Poland**	☐	☐	☐	☐	☐	☐
**Portugal**	☐	☐	☐	☐	☐	☐
**Sweden**	☐	☐	☐	☐	☐	☐
**Slovenia**	☐	☐	☐	☐	☐	☐
**Slovakia**	☐	☐	☐	☐	☐	☐

7. Please rate how closely you work with the following **LifeWatch Eric** nodes. *
Please make only one selection per row.

Tick all that apply.

	No Interaction	1	2	3	4	5
**Belgium**	☐	☐	☐	☐	☐	☐
**Bulgaria**	☐	☐	☐	☐	☐	☐
**Greece**	☐	☐	☐	☐	☐	☐
**Italy**	☐	☐	☐	☐	☐	☐
**Netherlands**	☐	☐	☐	☐	☐	☐
**Portugal**	☐	☐	☐	☐	☐	☐
**Slovenia**	☐	☐	☐	☐	☐	☐
**Spain (Head Office)**	☐	☐	☐	☐	☐	☐

Interaction with **Non-BioDT Consortium** Research Infrastructures

This section is optional but responses would be invaluable.

In this section, we are interested in how closely your national research infrastructure node/site (i.e., you and/or your team) collaborate with nodes/representatives of research infrastructures that aren't DiSSCo, eLTER, GBIF, or LifeWatch. We are interested in both international and national research infrastructures.

To facilitate sharing this information with us, we have prepared an excel sheet for you which can be downloaded here. Therein, you will find three columns:

- Name - the name of the research infrastructure node/site you collaborate with
- Nationality - the nationality of the research infrastructure node/site you collaborate with
- Collaboration - a number of 1-5 to indicate collaboration intensity as in the previous section

To avoid confusion of how to work with the 1-5 scale, here are some suggestions for how to link your collaboration to the levels in the scale:

1. You are present in group calls or mailing lists with the node/site in question
2. You interact with staff of the node/site in question sporadically
3. You collaborate on projects with the node/site in question
4. You collaborate on shared tasks with the node/site in question
5. You share offices and or staff with the node/site in question

NOTE: If you are a BioDT project partner, please answer as though you were not to avoid a strong biasing effect of this project on our understanding of the day-to-day makeup of the European biodiversity RI landscape.

Please forego entry of research infrastructures you know exist but don't interact with.

Once finished with the document, please edit its name to reflect your research infrastructure affiliation and nationality of your node/site and send it to erik.kusch@nhm.uio.no.

This content is neither created nor endorsed by Google.

Google Forms
